# Prevalence of Beijing Central Asian/Russian Cluster 94-32 among Multidrug-Resistant *M. tuberculosis* in Kazakhstan

**DOI:** 10.3390/antibiotics13010009

**Published:** 2023-12-20

**Authors:** Ainur Akhmetova, Venera Bismilda, Lyailya Chingissova, Maxim Filipenko, Ainur Akilzhanova, Ulan Kozhamkulov

**Affiliations:** 1 Laboratory of Genomic and Personalized Medicine, Center for Life Sciences, National Laboratory Astana, Nazarbayev University, Astana 010000, Kazakhstan; 2 Department of General Biology and Genomics, L.N. Gumilyov Eurasian National University, Astana 010000, Kazakhstan; 3 National Scientific Center of Phthisiopulmonology of the Republic of Kazakhstan, Almaty 050000, Kazakhstan; 4 Laboratory of Pharmacogenomics, Institute of Chemical Biology and Fundamental Medicine, Novosibirsk 630000, Russia; 5 Faculty of Natural Sciences, Novosibirsk State University, Novosibirsk 630090, Russia

**Keywords:** pulmonary tuberculosis, Beijing genotype, Central Asian/Russian type 94-32, MIRU-VNTR, multidrug-resistance

## Abstract

The Beijing genotype is the most distributed *M. tuberculosis* family in Kazakhstan. In this study, we identified dominant Beijing clusters in Kazakhstan and assessed their drug susceptibility profiles and association with the most widely spread mutation Ser531Leu of the *rpoB* gene and the mutation Ser315Thr of the *katG* gene associated with resistance to rifampicin and isoniazid, respectively. *M. tuberculosis* isolates (*n* = 540) from new TB cases were included in the study. MIRU-VNTR genotyping was performed for 540 clinical isolates to determine *M. tuberculosis* families using 24 loci. RD analysis was additionally performed for the Beijing isolates. The identification of mutations in the drug-resistance genes of *M. tuberculosis* was performed with allele-specific real-time PCR and Sanger sequencing. The Beijing genotype was identified in 60% (324/540) of the clinical isolates. Central Asian/Russian cluster 94-32 was the most distributed cluster among the Beijing isolates (50.3%; 163/324). Three other dominant Beijing clusters were identified as 94-33 (3.4%; 11/324), 100-32 (3.1%; 10/324) and 99-32 (3.1%; 10/324). The Beijing genotype was associated with drug-resistant TB *(p* < 0.0001), including multidrug-resistant TB *(p* < 0.0001), in our study. An association of the mutation Ser531Leu of the *rpoB* gene with the Beijing genotype was found (*p* < 0.0001; OR = 16.0000; 95%CI: 4.9161–52.0740). Among the Beijing isolates, cluster 94-32 showed an association with MDR-TB (*p* = 0.021). This is why the evaluation of the Beijing genotype and its clusters is needed to control MDR-TB in Kazakhstan.

## 1. Introduction

The Beijing genotype is the major component of the East Asian lineage (or lineage 2). Beijing strains were first described in 1995 by van Soolingen et al. [1]. Beijing isolates have been found in all continents of the world [2], and they are predominant in Asia (1.4–79.4%) and Eastern Europe (22–71%) [3]. Many studies have shown an association between the Beijing genotype and multidrug-resistant tuberculosis (MDR-TB), the most dangerous form of TB that is characterized by resistance to the most effective antibiotics used in TB treatment, rifampicin and isoniazid [4], and its massive spread [3]. Beijing MDR isolates were also identified during several TB outbreaks in the US [5] and were the cause of relatively recent outbreaks in some European countries [6,7].

Previous studies have shown that 95% of rifampicin-resistant *M. tuberculosis* isolates have mutations in the 81bp RRDR (rifampicin-resistance-determining region) of the *rpoB* gene between codons 507 and 533 [8]. The most commonly identified mutation among rifampicin-resistant isolates is the mutation with an amino acid substitution of serine to leucine, Ser531Leu, in codon 531 of the *rpoB* gene [9]. Among isoniazid-resistant *M. tuberculosis* isolates, in 50–95% of cases, a mutation with the amino acid substitution of serine to threonine, Ser315Thr, in codon 315 of the *katG* gene was found [10]. Mutations in the *fabG-inhA* promoter region were determined in 8–43% of cases [10]. The *oxyR-ahpC* promoter region mutations were detected among 10–18% of isoniazid-resistant isolates [10].

Beijing strains have a higher ability to resist TB treatment and are considered an essential risk factor for treatment failure or TB recurrence [11,12]. However, the elevated ability to acquire resistance to anti-TB drugs is not common for all isolates of the Beijing genotype; this differs among various Beijing subtypes and geographic regions [13,14].

Clinically and epidemiologically important Beijing genotype clusters were determined with an IS6110-RFLP (insertion sequence 6110–restriction fragment length polymorphism) and 24 MIRU-VNTR (mycobacterial interspersed repetitive units–variable number tandem repeats) approaches, i.e., the W strain, which was the cause of the MDR-TB outbreak in the US in the 1990s [5], Gran Canaria GC1237, which was the reason for the rapid distribution of TB in Spain in the 1990s [15] and two large clusters widely spread in former Soviet Union (FSU) countries—Central Asian/Russian cluster 94-32 and Russian cluster 100-32. Studies have revealed a strong association between Russian cluster 100-32 and MDR-TB. Isolates of this cluster spread 10 times quicker than other Beijing isolates [16]. Also, Beijing 94-32 and 100-32 isolates were found among drug-resistant samples in the US, Europe, and East Asia [16,17,18].

Other significant early ancient Beijing genotype clusters are 1071-32 and 14717-15. Various investigations have revealed that isolates of cluster 1071-32 are distributed in the Russian Federation, Armenia, Serbia, Albania, Greece and China [19], and these were isolated mostly from patients with MDR-TB, pre-extensively drug resistant (XDR)-TB or XDR-TB. Beijing 14717-15 isolates are hypervirulent and have been shown to be highly lethal in mouse models. Strains of this cluster were circulated in the Russian Federation, Korea and Lithuania [19].

In Kazakhstan, studies were carried out to investigate mutations in the *M. tuberculosis* genes responsible for drug resistance to various antibiotics [20,21]. Several studies were performed on the molecular genotyping [22,23] and whole-genome sequencing of *M. tuberculosis* [24,25,26]. However, Beijing genotype clusters and their association with mutations in drug-resistant genes have not been fully investigated.

This study aims to identify the dominant Beijing clusters among new TB cases in Kazakhstan and evaluate their drug susceptibility profiles and associations with the main mutations Ser531Leu in the *rpoB* gene and Ser315Thr in the *katG* gene associated with resistance to the most effective first-line antibiotics, rifampicin and isoniazid, respectively.

## 2. Results

### 2.1. Phenotypic Characteristics of M. tuberculosis Isolates

The results of the DST revealed the prevalence of drug-resistant clinical isolates (55.6%; 300/540) among the 540 *M. tuberculosis* samples collected from new TB cases in Kazakhstan. Among the 300 resistant TB forms, MDR-TB associated with the main antibiotics, rifampicin and isoniazid, was identified in 52.3% (157/300) of cases. Four combinations of drug resistance were determined among the MDR samples; 37% out of 52.3% (111/157) of the isolates with MDR displayed resistance to all four first-line antibiotics (rifampicin, isoniazid, ethambutol and streptomycin) that were tested (Table S1).

### 2.2. Evaluation of M. tuberculosis Families

Initially, 24 MIRU-VNTR genotyping was performed for 561 *M. tuberculosis* clinical isolates. A total of 21 (3.7%) of the isolates showed two bands among the 24 MIRU-VNTR loci, which means a double infection or mixed infection. For this reason, these isolates were further excluded from the study. 

According to results of the 24 MIRU-VNTR genotyping, 9 *M. tuberculosis* families were identified among the 540 clinical isolates from new TB cases in Kazakhstan. A total of 60% (324/540) of the isolates belonged to the Beijing family. The second most distributed *M. tuberculosis* family in Kazakhstan was LAM (Latin American Mediterranean) at 12.9% (70/540). Isolates of Ural and Haarlem families were found in 8.5% (46/540) and 5.4% (29/540) of the cases, respectively. Other *M. tuberculosis* families were determined in less than 5% of the cases: Cameroon—4.1% (22/540), New-1—3.7% (20/540), S—0.9% (5/540), Delhi/CAS—0.2% (1/540) and X—0.2% (1/540). The remaining 4.1% (22/540) of the isolates from Kazakhstan were not assigned to any known *M. tuberculosis* families in the MIRU-VNTRplus.org database. All 22 (4.1%) of the unknown isolates had unique MIRU-VNTR digital profiles.

### 2.3. Beijing Genotype Clusters in Kazakhstan

The results of the 24 MIRU-VNTR showed that the Beijing family was the most prevalent *M. tuberculosis* family among the clinical isolates obtained from the new TB cases in Kazakhstan (60%; 324/540). RD (region of difference) analysis revealed that all the 324 Beijing isolates had the RD105 deletion.

In 69.1% (224/324) of the cases, the Beijing isolates were drug-resistant (*p* < 0.0001), and 40.7% (132/224) of the Beijing samples revealed multidrug resistance (*p* < 0.0001). In 50.3% (163/324) of the cases, the Beijing type 94-32 was identified among all the Beijing isolates. Other frequently distributed types found among the Beijing isolates included 94-33 (3.4%; 11/324), 100-32 (3.1%; 10/324) and 99-32 (3.1%; 10/324).

The 24 MIRU-VNTR genotyping of the 324 Beijing *M. tuberculosis* clinical isolates displayed 90 variants of digital profiles. Twenty-three profiles were represented by clusters, which included from 2 to 163 isolates according to the MIRU-VNTR*plus* nomenclature (Table 1). A total of 79.3% (257/324) of the Beijing isolates were in clusters, while 20.7% (67/324) had unique digital MIRU-VNTR profiles found only in one isolate in our sample collection.

To characterize and determine the features of the most distributed Beijing genotype in Kazakhstan, we firstly identified the main clusters of the genotype found in Kazakhstan and their drug susceptibility profiles. Further, we analyzed the distribution of various clusters of the Beijing genotype in different drug susceptibility groups (drug-resistant vs. drug-susceptible isolates and MDR vs. other drug-resistant (DR) clinical samples, which included mono-/poly-resistant isolates) in comparison with other Beijing isolates combined into one group.

The most clustered clinical samples (63.4%; 163/257) were assigned to the Central Asian/Russian type 94-32. The remaining three dominant clusters of the Beijing genotype, 94-33 (4.3%; *n* = 11), 100-32 (3.8%; *n* = 10) and 99-32 (3.8%; *n* = 10), were found in 11.9% (*n* = 31) of patients (Table 1).

Other clusters were identified in less than 3% of the cases. Clusters 96-32 and 95-32 each consisted of seven (2.7%) isolates. MLVA MtbC 15-9 types 94-15 and 96-145 had six (2.3%) clinical samples. Five (1.9%) isolates were included in cluster 97-32. Four clusters (1048-32, 7308-32, 1075-32 and 7553-145) each consisted of three (1.2%) isolates. Ten clusters (11427-32, 9344-32, 9343-32, 1068-32, 95-33, 809-32, 9342-32, 94-554, 94-145 and ?-32) each had two (0.8%) isolates (Table 1).

A total of 71.2% (183/257) of the clustered isolates of the Beijing genotype showed one of the resistant forms of TB (mono-/poly-resistant and multidrug-resistant TB). The remaining 28.8% (74/257) of the samples were drug-susceptible to the first-line anti-TB drugs that were tested (Table 1). Among the largest Central Asian/Russian cluster 94-32 with the MIRU-VNTR profile 244233352644425153353823, the isolates were mainly resistant to first-line antibiotics (73%; 119/163). The association of cluster 94-32 of the Beijing genotype with drug-resistant forms of TB was not statistically significant (*p* = 0.149). However, the cluster 94-32 samples were mostly isolated from patients with MDR-TB rather than other drug-resistant TB forms (66.4%; 79/119 vs. 33.6%; 40/119) among the antibiotic-resistant *M. tuberculosis* samples and showed statistically a significant association (*p* = 0.021). Among the other Beijing isolates grouped into one group, MDR and mono-/poly-resistant isolates were found almost in equal quantities (50.5%; 53/105 vs. 49.5%; 52/105) (Table 2).

A comparative analysis of the data also revealed that three other main clusters, 99-32, 94-33 and 100-32, of the Beijing genotype were not associated with infection by drug-resistant TB and any form of resistant TB (Table 2).

The number of drug-resistant and susceptible Beijing isolates were equal in clusters 99-32, 9343-32, 1068-32 and 809-32. The dominance of drug-resistant isolates compared to drug-susceptible isolates was noted in clusters 100-32 (8/10; 80% vs. 2/10; 20%) and 96-32 (4/7; 57.1% vs. 3/7; 42.9%). In three clusters, 9344-32 (*n* = 2), 1068-32 (*n* = 1) and 7308-32 (*n* = 1), the isolates were MDR only (100%), while in clusters 9343-32 (*n* = 1), 1048-32 (*n* = 3) and 809-32 (*n* = 1), the samples were mono-/poly-resistant only (100%). A total of 34 isolates in 11 clusters (11427-32, 9344-32, 1048-32, 95-33, 9342-32, 95-32, 1075-32, 96-145, 7553-145, 94-554 and 94-145) were 100% resistant to first-line antibiotics (Table 1). However, a statistical analysis of the different clusters of the Beijing genotype mentioned in this paragraph with various forms of drug resistance did not reveal any significant association of a specific Beijing cluster with drug resistance, including MDR-TB and TB with mono-/poly-resistance (*p* > 0.05). It is important to mention that the Russian cluster 100-32, which was shown to be associated with MDR-TB [27], did not correlate with resistant TB, including MDR-TB, in our study (Table 2).

Drug-susceptible isolates of the Beijing genotype prevailed among clusters 97-32 (60%; 3/5), 94-15 (66.7%; 4/6), 94-33 (54.5%; 6/11) and 7308-32 (66.7%; 2/3) in comparison with the antibiotic-resistant isolates. However, the statistical analysis did not show any significant association of these clusters with drug-susceptible TB (*p* > 0.05).

### 2.4. Determination of Mutations in katG, fabG-inhA, oxyR-ahpC and rpoB Genes of M. tuberculosis Clinical Isolates

The most common mutations in the *rpoB* (Ser531Leu in codon 531) and the *katG* (Ser315Thr in codon 315) genes of 540 *M. tuberculosis* isolates associated with resistance to rifampicin and isoniazid, respectively, were analyzed by allele-specific real-time PCR (AS-RT-PCR).

The results of the AS-RT-PCR displayed that among the rifampicin-resistant isolates (*n* = 175), the Ser531Leu mutation of *rpoB* was found in 87.4% (153/175) of the cases. The remaining 12.6% (22/175) resistant isolates did not show the mutation (Table 3). Among the rifampicin-susceptible isolates (*n* = 365) in 97.8% (357/365) of the cases, no Ser531Leu mutation was determined in the gene. The presence of the Ser531Leu mutation in the *rpoB* gene was detected among the rest of the eight (2.2%) susceptible isolates.

DNA sequencing of the *rpoB* gene of 22 (12.6%) rifampicin-resistant isolates without Ser531Leu mutation showed mutations in three codons of the *rpoB* gene (Table 4). Three and two variants of the mutations were determined in codons 526 and 531 among all resistant isolates, respectively. The His526Leu mutation in codon 526 was identified in 5.1% (*n* = 9) of the cases. Two other mutations were detected in codon 526 of the *rpoB* gene of two isolates: His526Pro (0.6%; *n* = 1) and His526Tyr (0.6%; *n* = 1), respectively. In codon 531, two variants of mutations with amino acid substitutions of serine to phenylalanine Ser531Phe (0.6%; *n* = 1) and serine to tryptophan Ser531Trp (0.6%; *n* = 1) were found in two isolates. An amino acid change from leucine to proline was identified in codon 533 (Leu533Pro) of the *rpoB* gene in two (1.1%) isolates. In 4% (*n* = 7) of the cases, any mutations in the *rpoB* gene were not noted. Among 2.2% (*n* = 8) of the rifampicin-susceptible strains, the Ser531Leu mutation of the *rpoB* gene was confirmed by Sanger sequencing.

The statistical analysis of the obtained data revealed that among the *M. tuberculosis* rifampicin-resistant clinical isolates with confirmed mutations in different codons of the *rpoB* gene (*n* = 177), there was a significant association between the Beijing genotype and the presence of the most common mutation Ser531Leu in codon 531 of the *rpoB* gene (*p* < 0.0001; OR = 16.0000; 95% CI: 4.9161–52.0740) (Table 5).

The statistical analysis of the predominant Central Asian/Russian cluster 94-32 of the Beijing genotype in our sample collection did not show any association with the Ser531Leu mutation of the *rpoB* gene, as this mutation was detected in almost identical quantities among the isolates of cluster 94-32 and the other Beijing isolates pooled together (97.7% vs. 95.2%; *p* = 0.4066; OR = 2.1610; 95% CI: 0.3502–13.3347). The comparative analysis of the samples in the other prevalent clusters 100-32, 99-32 and 94-33 with the other Beijing isolates separately did not display an association with Ser531Leu mutation of the *rpoB* gene (Table 5).

The rifampicin-resistant isolates of the Beijing genotype had a strong association with the Ser531Leu mutation in codon 531 of the *rpoB* gene (*p* < 0.0001; OR = 16.0000; 95% CI: 4.9161–52.0740). It is interesting to note that the rifampicin-resistant isolates of the LAM (Latin American Mediterranean) genotype had a significant association with the His526Leu mutation in codon 526 of the *rpoB* gene (*p* < 0.0001; OR = 141.3333; 95% CI: 15.9020–1256.1369). In general, among the non-Beijing group, the LAM genotype revealed a strong correlation with drug-resistant TB (*p* < 0.0001).

The AS-RT-PCR results showed that the most common mutation with the amino acid change from serine to threonine Ser315Thr in codon 315 of the *katG* gene was identified in 96.5% (220/228) of the cases among the isoniazid-resistant isolates. The presence of the Ser315Thr mutation in the *katG* gene was not observed in the other eight (3.5%) isolates (Table 3). Among the 312 isoniazid-susceptible isolates, in 95.8% (299/312) of the cases, the most distributed mutation Ser315Leu of the *katG* gene was not detected, and in 4.2% of the cases (13/312), the clinical isolates had the mutation.

Further, to identify other mutations in the genes that play an important role in the formation of resistance to isoniazid, Sanger sequencing of the *katG*, *fabG-inhA* and *oxyR-ahpC* genes of eight resistant isolates without the Ser315Thr mutation was carried out. The sequencing results revealed that 0.9% (*n* = 2) of the samples had a mutation in the *fabG-inhA* promoter region (15 C-T). The other 2.6% (*n* = 6) of the isolates showed no mutations in the *katG*, *fabG-inhA* and *oxyR-ahpC* genes (Table 6). The presence of the Ser315Thr mutation in the *katG* gene of 13 isoniazid-susceptible isolates was confirmed by Sanger sequencing. Sequencing of the *fabG-inhA* and *oxyR-ahpC* promoter regions of these 13 isoniazid-susceptible samples did not display any changes.

The comparative analysis showed that among the isoniazid-resistant isolates with confirmed mutations in the *katG* or the *fabG-inhA* genes (*n* = 235), over 97% of the samples had the Ser315Thr mutation in the *katG* gene, regardless of whether they belonged to the Beijing genotype or non-Beijing genotypes of *M. tuberculosis* (*p* = 0.2603; OR = 4.9744; 95% CI: 0.3046–81.2405). In terms of the Beijing genotype clusters (*n* = 195), a similar trend was identified to that described above. Clusters 94-32, 100-32, 99-32 and 94-33, in comparison with the other Beijing isolates individually, did not have an association with the main mutation Ser315Thr in codon 315 of the *katG* gene, as almost an equal number of samples in all the cluster groups had this mutation (Table 5).

## 3. Discussion

Distribution of drug-resistant TB forms, including rifampicin-resistant TB (RR-TB), that is resistant to the main first-line antibiotic rifampicin and MDR-TB (resistant to rifampicin and isoniazid), the most dangerous form of TB remains one of the critical issues of the healthcare system in Kazakhstan. According to a World Health Organization (WHO) report, Kazakhstan is on the list of 30 countries with high rates of MDR/RR-TB. In 2021, 3.1% more cases of MDR/RR-TB (*n* = 450,000) were registered in the world compared to 2020, and >50% of the MDR/RR-TB cases among previously treated patients were detected from FSU countries [28]. Therefore, it is important to investigate the most distributed *M. tuberculosis* genotypes and their clusters in Kazakhstan as well as to monitor their spread and apply adequate control measures to combat tuberculosis.

This study revealed that 60% of the *M. tuberculosis* clinical isolates among the new TB (324/540) cases in Kazakhstan belonged to the Beijing genotype. All the Beijing isolates (*n* = 324) showed the deletion of the RD105 locus. Nowadays, the RD105 deletion serves as a marker of the Beijing genotype [29] and is used for the identification of Beijing isolates [30]. Beijing type 94-32 was predominant among the Beijing isolates and was found in 50.3% (163/324) of the cases, followed by types 94-33 (3.4%; 11/324), 100-32 (3.1%; 10/324) and 99-32 (3.1%; 10/324).

The prevalence of the Beijing family isolates among *M. tuberculosis* samples from Kazakhstan was also noted in earlier studies. The Beijing genotype was found in 65.3% (177/271) of cases among isolates from three regions of Kazakhstan (South and North Kazakhstan and Almaty city) [22]. Among the isolates resistant and susceptible to pyrazinamide, Beijing family strains were identified in 78.4% (58/74) of the cases [20]. In a study by Skiba et al., 72.2% (109/151) of the isolates belonged to the Beijing family, and 101 out of 109 Beijing isolates were grouped as 94-32 and related types [23]. In a study by Hillemann et al., the Beijing genotype was identified in 64.1% (59/92) of cases among MDR isolates and in 64% (32/50) of cases among isoniazid- but not rifampicin-resistant (INH^r^/RMP^s^) strains [21]. The study revealed an association of the Ser531Leu mutation of the *rpoB* gene with the Beijing genotype among the MDR group (*p* = 0.027) and an association of the Ser315Thr mutation of the *katG* gene among the INH^r^/RMP^s^ group (*p* = 0.012). In the present study, the Beijing genotype clusters distributed in Kazakhstan were described, their drug susceptibility profiles and correlation with the main mutations Ser531Leu in the *rpoB* gene and Ser315Thr in the *katG* gene associated with resistance to the most effective first-line antibiotics, rifampicin and isoniazid, respectively, were investigated.

In our study, the Beijing genotype was associated with drug-resistant TB compared to drug-susceptible TB (*p* < 0.0001). Among the drug-resistant *M. tuberculosis* clinical isolates, the Beijing genotype had a significant association with MDR-TB compared to TB with other DR (mono-/poly-resistance) (*p* < 0.0001). In previous studies conducted in Kazakhstan, the Beijing genotype also showed an association with infection by drug-resistant TB, including MDR-TB [23].

In our research, drug-resistant TB slightly prevailed among the isolates of the Central Asian/Russian cluster 94-32 compared to the other Beijing isolates pooled together (73% vs. 65.2%; *p* = 0.149). However, the Beijing cluster 94-32 was found almost two times more among MDR-TB patients than TB patients with other DR (66.4% vs. 33.6%; *p* = 0.021). Other Beijing clusters, including the prevalent Beijing clusters (94-33, 100-32 and 99-32) found in this study, did not correlate with drug-resistant TB and MDR-TB. In the earlier study by Skiba et al. [23], association of MDR-TB with cluster 94-32 was not confirmed (*p* = 0.07).

The Central Asian/Russian type 94-32 is highly dominant in Central Asian countries [31] and the Russian Federation [16]. The Russian cluster 100-32 is associated with MDR-TB and is widely distributed in different regions of Russia [27]. The cluster 100-32 is one of the predominant clusters found in Central Asia and Eastern Europe [3,31]. According to a report from the European Center for Disease Prevention and Control (2016), cluster 94-32 was determined among 23.7% of MDR-TB patients from 14 countries in Europe, and the Russian cluster 100-32 was detected among 33.6% of individuals with MDR-TB from 11 European countries [17]. The Beijing 94-32 and 100-32 clusters were also identified in immigrants from FSU countries in the US [16]. Among Central Asian countries (Kyrgyzstan, Tajikistan and Uzbekistan), the Beijing cluster 94-32 had an identical drug resistance profile as other Beijing isolates, while the Beijing cluster 100-32, in 99.3% (66/70) of cases, was MDR, pre-XDR or XDR [31]. The Beijing clusters 94-33 and 99-32 were found in FSU countries [32,33].

In our study, 4.1% (22/540) of the clinical isolates were classified as ‘unknown’, as they were not linked to any known *M. tuberculosis* families. Isolates with ‘undefined’ families were also found in other investigations of Central Asian isolates in 0.7–5.3% of cases [23,31].

Among the rifampicin-resistant *M. tuberculosis* isolates (*n* = 175), mutations in three codons of the *rpoB* gene were identified in 96% (168/175) of the cases. The main part of the resistant isolates (87.4%; 153/175) revealed the most commonly reported Ser531Leu mutation of the *rpoB* gene. This Ser531Leu mutation was significantly associated with the Beijing genotype in our study (*p* < 0.0001; OR = 16.0000; 95%CI: 4.9161–52.0740). An association of the Central Asian/Russian cluster 94-32 and three other dominant Beijing clusters (94-33, 100-32 and 99-32) with the Ser531Leu mutation of the *rpoB* gene was not found.

LAM genotype among the non-Beijing isolates was associated with drug resistance (*p* < 0.0001). Among the rifampicin-resistant isolates, the LAM genotype was associated with the His526Leu mutation of the *rpoB* gene (*p* < 0.0001; OR = 141.3333; 95%CI: 15.9020–1256.1369). This mutation of the *rpoB* gene was also identified in the genomes of drug-resistant LAM isolates (66.7%; 2/3) from Kazakhstan [34].

Among the isoniazid-resistant clinical isolates (*n* = 228), mutations in the *katG* and the *fabG-inhA* genes were determined in 97.4% (222/228) of the cases. The majority of the samples (96.5%; 220/228) had the most common Ser315Thr mutation in codon 315 of the *katG* gene. The association of the Ser315Thr mutation of the *katG* gene with the Beijing genotype and its clusters were not observed in our research.

The Ser531Leu mutation of the *rpoB* gene was associated with the Beijing genotype among MDR-TB (*p* = 0.027) cases in Kazakhstan in an earlier study [21], and the Ser315Thr mutation of the *katG* gene was shown to have an association with isoniazid-resistant (but rifampicin-susceptible) Beijing isolates (*p* = 0.012). However, in East Asian countries, the number of isolates with the most prevalent mutations, Ser315Thr in the *katG* gene and Ser531Leu in the *rpoB* gene, were similar between Beijing and non-Beijing groups [35].

In 4% (7/175) and 2.6% (6/228) of the cases, the rifampicin- and isoniazid-resistant clinical isolates did not reveal any mutations in the *rpoB* and *katG, fabG-inhA, oxyR-ahpC* genes, respectively. The absence of mutations in specific drug-resistant genes has been recorded in 2.3–10.38% of cases in various studies [36,37,38,39]. Mutations in other genes may cause resistance to anti-TB drugs in these resistant isolates. For example, whole-genome sequencing data of *M. tuberculosis* isolates from high TB-burden countries showed 23 novel *katG* mutations in isoniazid-resistant isolates [40] and mutations outside of the *rpoB* gene in rifampicin-resistant isolates [41,42]. Yet, genomic data of MDR, pre-XDR and XDR strains from Kazakhstan identified new genetic variants that may play an important role in the formation of drug resistance [24,25,41].

In addition, 2.2% (8/365) of the rifampicin- and 4.2% (13/312) of the isoniazid-susceptible isolates in this work showed the most common mutations, Ser531Leu of the *rpoB* gene and Ser315Thr of the *katG* gene, respectively. These isolates may have a low resistance to rifampicin and isoniazid [43].

## 4. Materials and Methods

### 4.1. Clinical Isolates of M. tuberculosis and DST

Clinical isolates of *M. tuberculosis* (*n* = 561) were collected from new cases of pulmonary tuberculosis from different oblasts of Kazakhstan. All the microbiological investigations, including the identification and isolation of *M. tuberculosis* cultures from the sputum of TB patients, were performed at the National Reference Laboratory of the National Scientific Center of Phthisiopulmonology (Almaty city). Drug susceptibility testing (DST) was carried out for four first-line anti-TB drugs by a microbiological method using a BACTEC-Mycobacterial Growth Indicator Tube (MGIT) 960 (BD Diagnostic System, Franklin Lakes, NJ, USA) with critical drug concentrations of 0.5 μg/mL for rifampicin, 0.1 μg/mL for isoniazid, 5 μg/mL for ethambutol and 1 μg/mL for streptomycin.

### 4.2. Genotyping

DNA of *M. tuberculosis* samples was isolated according to a standard protocol as described earlier [44].

The 24 MIRU-VNTR genotyping was performed for the *M. tuberculosis* clinical isolates as shown previously [22,45] to identify Beijing and non-Beijing *M. tuberculosis* isolates. The obtained MIRU-VNTR profiles were analyzed using the www.miru-vntrplus.org web resource (https://www.miru-vntrplus.org/MIRU/index.faces, accessed on 1 June 2023), where MLVA types (clusters) were assigned to the isolates. Two or more isolates with identical MIRU-VNTR profiles in a phylogenetic group were considered as a cluster [45].

The Beijing family isolates were additionally identified by RD analysis. Determination of RD105 deletion was conducted as shown previously [29,46]. Typing was carried out by real-time PCR using SYBR green PCR master mix (Applied Biosystems, Waltham, MA, USA) in a CFX96 Real-time System (Bio-Rad, Hercules, CA, USA) with an initial denaturation step of 95 °C for 3 min, followed by 41 cycles of denaturation at 95 °C for 5 s, annealing at 60 °C for 8 s and elongation at 72 °C for 10 s. Data acquisition was carried out on the SYBR channel, and melting curves of the amplification products were obtained from 72 °C to 97 °C (in 0.5 °C increments).

### 4.3. Detection of Mutations in Drug-Resistant Genes

Allele-specific real-time PCR (AS-RT-PCR) was performed for 540 clinical isolates for screening of the most distributed mutations, Ser531Leu in codon 531 of the *rpoB* gene and Ser315Thr in codon 315 of the *katG* gene, associated with drug resistance to rifampicin and isoniazid, respectively. AS-RT-PCR was carried out using SYBR green PCR master mix (Applied Biosystems) with similar PCR conditions to those used for the RD105 locus. The PCR reaction was performed with the allele-specific and reference primers (Table S2). The following isolates with confirmed by the Sanger sequencing results were used as controls: 2 rifampicin-resistant isolates with the Ser531Leu mutation and 2 isoniazid-resistant isolates with the Ser315Thr mutation as well as 2 rifampicin-susceptible and 2 isoniazid-susceptible isolates. Sterile mQ water was used as a negative control.

Sanger sequencing of the *rpoB* and *fabG-inhA, oxyR-ahpC, katG* genes associated with rifampicin and isoniazid, respectively, was performed for the drug-resistant or drug-susceptible samples where the results of AS-RT-PCR did not match the DST results. DNA sequencing of the *rpoB* (*n* = 30), *fabG-inhA* (*n* = 21), *oxyR-ahpC* (*n* = 21) and *katG* (*n* = 21) genes was carried out as described previously [47].

### 4.4. Statistical Analysis

IBM SPSS Statistics 24.0 and Medcalc online [48] were used to carry out the statistical analysis of the obtained data. The chi-squared test (χ^2^) and Fisher’s exact test were used to detect any significant differences between the two groups. Yates’ corrected χ^2^ and *p*-values were calculated with a 95% confidence interval (CI) for the mean where necessary. The results were considered statistically significant if the *p*-value was equal to or less than 0.05.

## 5. Conclusions

The Beijing genotype is the main *M. tuberculosis* genotype spread in Kazakhstan. The genotype correlates with the presence of the Ser531Leu mutation in codon 531 of the *rpoB* gene, which is associated with resistance to the most effective first-line anti-TB drug, rifampicin. Clusters 94-32, 94-33, 100-32 and 99-32 were the predominant clusters of the Beijing genotype among the new TB cases. The data obtained from this study revealed the essential role of the Beijing genotype and its Central Asian/Russian cluster 94-32 in the distribution of drug-resistant TB, particularly MDR-TB, in Kazakhstan. Therefore, necessary measures need to be taken to combat this epidemic. It is important to pay attention to the investigation and control of the Beijing genotype and its clusters for the monitoring of drug-resistant, especially multidrug-resistant, *M. tuberculosis* circulating in Kazakhstan and Central Asian countries.

## Figures and Tables

**Table 1:** Drug susceptibility of Beijing genotype clusters (two or more isolates) among new TB cases.

MLVA MtbC 15-9 Types	24 MIRU-VNTR Profile	Multidrug-Resistant Isolates (%)	Mono-/Poly-Resistant Isolates (%)	Drug-Resistant Isolates (%)	Susceptible Isolates (%)	Total (%)
94-32	244233352644425153353823	79 (48.5)	40 (24.5)	119 (73)	44 (27)	163 (63.4)
99-32	244233352644425153353723	3 (30)	2 (20)	5 (50)	5 (50)	10 (3.8)
11427-32	244233352644425153353523	1 (50)	1 (50)	2 (100)	0	2 (0.8)
9344-32	245233352644425153353823	2 (100)	0	2 (100)	0	2 (0.8)
97-32	244233332644425153353823	1 (20)	1 (20)	2 (40)	3 (60)	5 (1.9)
9343-32	244234352644425153353823	0	1 (50)	1 (50)	1 (50)	2 (0.8)
1068-32	244233352644425143353823	1 (50)	0	1 (50)	1 (50)	2 (0.8)
1048-32	244233352644425173353823	0	3 (100)	3 (100)	0	3 (1.2)
94-15	244233352644425153353822	1 (16.66)	1 (16.66)	2 (33.3)	4 (66.7)	6 (2.3)
94-33	244233352644425153353824	1 (9.1)	4 (36.4)	5 (45.5)	6 (54.5)	11 (4.3)
95-33	244233352634425153353824	1 (50)	1 (50)	2 (100)	0	2 (0.8)
7308-32	244232352644425153353823	1 (33.3)	0	1 (33.3)	2 (66.7)	3 (1.2)
809-32	244232352634425153353823	0	1 (50)	1 (50)	1 (50)	2 (0.8)
9342-32	244235352644425153353823	1 (50)	1 (50)	2 (100)	0	2 (0.8)
95-32	244233352634425153353823	5 (71.4)	2 (28.6)	7 (100)	0	7 (2.7)
1075-32	244233352644425163353723	2 (66.7)	1 (33.3)	3 (100)	0	3 (1.2)
96-32	244233362644425153353823	1 (14.2)	3 (42.9)	4 (57.1)	3 (42.9)	7 (2.7)
96-145	244233361644425153353823	4 (66.7)	2 (33.3)	6 (100)	0	6 (2.3)
7553-145	244233361644425153343823	2 (66.7)	1 (33.3)	3 (100)	0	3 (1.2)
94-554	244233351644425153353824	1 (50)	1 (50)	2 (100)	0	2 (0.8)
94-145	244233351644425153353823	1 (50)	1 (50)	2 (100)	0	2 (0.8)
100-32	244233352644425173353723	4 (40)	4 (40)	8 (80)	2 (20)	10 (3.8)
?-32	244232352544425153343823	0	0	0	2 (100)	2 (0.8)
Total		112 (43.6)	71 (27.6)	183 (71.2)	74 (28.8)	257 (100)

**Table 2:** Association analysis of the relationship between different *M. tuberculosis* genotypes, including Beijing isolates with drug resistance among new TB cases.

Genotypes/Beijing Clusters	Resistant TB	Susceptible TB	OR	95% CI	*p*	MDR	Other DR	OR	95% CI	*p*
Beijing Non-Beijing	224 (69.1%) 76 (35.2%)	100 (30.9%) 140 (64.8%)	-	-	*p* < 0.0001 *	132 (59%) 25 (32.9%)	92 (41%) 51 (67.1%)	-	-	*p* < 0.0001 *
94-32 Other Beijing	119 (73%) 105 (65.2%)	44 (27%) 56 (34.8%)	-	-	*p* = 0.149	79 (66.4%) 53 (50.5%)	40 (33.6%) 52 (49.5%)	-	-	*p* = 0.021 *
100-32 Other Beijing	8 (80%) 216 (68.8%)	2 (20%) 98 (31.2)	1.8148	0.3784–8.7040	*p* = 0.4562	4 (50%) 128 (59.3%)	4 (50%) 88 (40.7%)	0.6875	0.1675–2.8223	*p* = 0.6031
99-32 Other Beijing	5 (50%) 219 (69.7%)	5 (50%) 95 (30.3%)	0.4338	0.1227–1.5336	*p* = 0.1949	3 (60%) 129 (58.9%)	2 (40%) 90 (41.1%)	1.0465	0.1714–6.3906	*p* = 0.9607
94-33 Other Beijing	5 (45.5%) 219 (70%)	6 (54.5%) 94 (30%)	0.3577	0.1065–1.2009	*p* = 0.0962	1 (20%) 131 (59.8%)	4 (80%) 88 (40.2%)	0.1679	0.0185–1.5277	*p* = 0.1132

* Statistically significant result; these values were not calculated in SPSS.

**Table 3:** Determination of Ser531Leu mutation in the *rpoB* gene and Ser315Thr mutation in the *katG* gene of clinical isolates of *M. tuberculosis*.

Rifampicin	Number of Isolates	Ser531Leu Mutation		Isoniazid	Number of Isolates	Ser315Thr Mutation	
		Yes	No			Yes	No
Resistant isolates	175 (100%)	153 (87.4%)	22 (12.6%)	Resistant isolates	228 (100%)	220 (96.5%)	8 (3.5%)
Susceptible isolates	365 (100%)	8 (2.2%)	357 (97.8%)	Susceptible isolates	312 (100%)	13 (4.2%)	299 (95.8%)
Total:	540 (100%)	161 (29.8%)	379 (70.2%)	Total:	540 (100%)	233 (43.1%)	307 (56.9%)

**Table 4:** Variants of amino acid substitutions in the *rpoB* gene of clinical isolates of *M. tuberculosis*.

Rifampicin	*rpoB* Gene	Mutation	Number of Isolates
Resistant isolates	526 codon	His526Leu His526Tyr His526Pro	9 (5.1%) 1 (0.6%) 1 (0.6%)
	531 codon	Ser531Phe Ser531Trp	1 (0.6%) 1 (0.6%)
	533 codon	Leu533Pro	2 (1.1%)
	no mutation	no mutation	7 (4%)
Total:			22 (12.6%)
Susceptible isolates	531 codon	Ser531Leu	8 (2.2%)
Total:			8 (2.2%)

**Table 5:** Statistical analysis of the association between different *M. tuberculosis* genotypes, including Beijing isolates with mutations in the *rpoB* and the *katG* genes among new TB cases.

Genotypes/Beijing Clusters	Ser531Leu	Other Mutations	OR	95% CI	*p*	Ser315Thr	Other Mutations	OR	95% CI	*p*
Beijing Non-Beijing	144 (96.6%) 18 (64.3%)	5 (3.4%) 10 (35.7%)	16.0000	4.9161–52.0740	*p* < 0.0001 *	194 (99.5%) 39 (97.5%)	1 (0.5%) 1 (2.5%)	4.9744	0.3046–81.2405	*p* = 0.2603
94-32 Other Beijing	85 (97.7%) 59 (95.2%)	2 (2.3%) 3 (4.8%)	2.1610	0.3502–13.3347	*p* = 0.4066	106 (99.1%) 88 (100%)	1 (0.9%) 0	0.4011	0.0161–9.9701	*p* = 0.5774
100-32 Other Beijing	4 (80%) 140 (97.2%)	1 (20%) 4 (2.8%)	0.1143	0.0103–1.2676	*p* = 0.0773	9 (100%) 185 (99.5%)	0 1 (0.5%)	0.1536	0.0059–4.0279	*p* = 0.2610
99-32 Other Beijing	3 (100%) 141 (96.6%)	0 5 (3.4%)	0.2721	0.0125–5.9394	*p* = 0.4080	4 (100%) 190 (99.5%)	0 1 (0.5%)	0.0709	0.0025–1.9886	*p* = 0.1197
94-33 Other Beijing	3 (100%) 141 (96.6%)	0 5 (3.4%)	0.2721	0.0125–5.9394	*p* = 0.4080	6 (100%) 188 (99.5%)	0 1 (0.5%)	0.1034	0.0038–2.7902	*p* = 0.1771

* Statistically significant result; these values were not calculated in SPSS.

**Table 6:** Mutations in genes of *M. tuberculosis* clinical isolates associated with resistance to isoniazid.

Isoniazid	Genes	Mutation	Number of Isolates
Resistant isolates	*fabG-inhA*	15 C-T	2 (0.9%)
	No mutation	No mutation	6 (2.6%)
Total:			8 (3.5%)
Susceptible isolates	*katG*	Ser315Thr	13 (4.2%)
Total:			13 (4.2%)

## Data Availability

The data presented in this study are available on request from the corresponding authors.

## Supplementary Materials

The following supporting information can be downloaded at: https://www.mdpi.com/article/10.3390/antibiotics13010009/s1, Table S1: Description of *M. tuberculosis* clinical isolates resistant to antituberculosis drugs; Table S2: Sequences of primers used in AS-RT-PCR to determine resistance in the analyzed *M. tuberculosis* genes.
