# Supplementary material for: Prevalence of Beijing Central Asian/Russian Cluster 94-32 among Multidrug-Resistant M. tuberculosis in Kazakhstan
Source: Antibiotics (Basel). 2023 Dec 20;13(1):9. doi: 10.3390/antibiotics13010009 (PMC10812519; doi:10.3390/antibiotics13010009)
Supplement: Supplementary file 1 [file antibiotics-13-00009-s001.zip › antibiotics-2748047-supplementary.pdf]

Table S1. Description of *M. tuberculosis* clinical isolates resistant to antituberculosis drugs

Drug-resistant *M. tuberculosis* clinical isolates	n (%)
Monoresistant isolates (resistant only to one anti-TB drug):	*49 (16.3%)*
resistant only to RMP*	6 (2%)
resistant only to INH*	14 (4.7%)
resistant only to EMB*	7 (2.3%)
resistant only to SM*	22 (7.3%)
Multidrug resistant isolates (resistant at least to RMP and INH):	*157 (52.3%)*
resistant to RMP+INH	13 (4.3%)
resistant to RMP+INH+EMB	3 (1%)
resistant to RMP+INH+SM	30 (10%)
resistant to RMP+INH+EMB+SM	111 (37%)
Polyresistant isolates (resistant at least to two or more antibiotics except RMP+INH combination):	*94 (31.3%)*
resistant to INH+EMB	4 (1.33%)
resistant to INH+SM	25 (8.32%)
resistant to INH+EMB+SM	28 (9.32%)
resistant to RMP+EMB	1 (0.33%)
resistant to RMP+SM	10 (3.32%)
resistant to RMP+EMB+SM	1 (0.33%)
resistant to EMB+SM	25 (8.32%)
Total:	*300 (100%)*

*RMP – rifampicin; INH – isoniazid; EMB – ethambutol; SM – streptomycin.

Table S2. Sequences of primers used in AS-RT-PCR to determine resistance in the analyzed *M. tuberculosis* genes

Genetic locus	Codon	Primer name	Nucleotide sequence (5′-3′)
katG	315	Mt315F	GTAAGGACGCGATCACCAC
		Wt315F	GTAAGGACGCGATCACCAG
		315R	GAAACTGTTGTCCCATTTCG
rpoB	531	Mt531F	CACAAGCGCCGACTGTT
		Wt531F	CACAAGCGCCGACTGTC
		531R	GGCACGCTCACGTGACAGAC
